# Diagnostic performance of central and generalized adiposity in detecting risk of diabetes mellitus in adolescents

**DOI:** 10.4314/ahs.v22i4.15

**Published:** 2022-12

**Authors:** Danladi Ibrahim Musa, Busayo E Agbana, Moses F Adeola, Benjamin M Idache, Sunday Abu, Tochi Emmanuel Iwuagwu

**Affiliations:** 1 Department of Human Kinetics and Health Education, Kogi State University, Anyigba 272102, Nigeria; 2 Department of Community Medicine, College of Health Sciences, Kogi State University, Anyigba 272102, Nigeria; 3 Department of Human Kinetics and Health Education, University of Nigeria, Nsukka 970101, Nigeria

**Keywords:** Abdominal obesity, adolescents, anthropometry, ROC curve, T2DM

## Abstract

**Background:**

The prevalence of type 2 diabetes mellitus (T2DM) is increasing in all age groups, including the adolescent globally.

**Objective:**

This study examined the association of adiposity with the risk of T2DM in adolescents aged 11 to 19 years.

**Methods:**

This study comprised 403 adolescent boys (202) and girls (201). Participants were assessed in three indices of body fat and fasting blood glucose (FBG). Multivariate regression models assessing the associations of the independent variables with risk of T2DM were conducted. Receiver operating characteristic curve (ROC) analysis was used to determine the predictive capacities of the body fat proxies to detect risk of T2DM.

**Results:**

The prevalence of glucose abnormalities was 13.6% and 1.8% for pre-diabetes and diabetes respectively. Among the body fat indices in boys, WHtR was the only independent predictor (*β* =0.599, p<0.001) of T2DM risk. Both the WHtR and WC had significant (p<0.001) Areas under curve (AUC), with WHtR as the best body fat indicator for identifying risk of T2DM in boys. The independent variables had no discriminatory capacities to detect T2DM risk in girls.

**Conclusions:**

This study has demonstrated that central fat is more important than total fat in detecting risk of T2DM in Nigerian adolescent boys.

## Introduction

Type 2 diabetes mellitus (T2DM) is a complex and chronic metabolic disease with risk factors spanning behavioural, genetic and social dimensions, and it is associated with serious complications and co-morbidities, including cardiovascular disease (CVD), metabolic syndrome (MS), non-alcoholic fatty liver disease, osteoarthritis, end-stage kidney disease, retinopathy and limb amputation^1^. The World Health Organization^2^ predicted that there would be at least 350 million people in the world with T2DM by 2030. Until recently, T2DM was regarded as a disease of the middle age and adults, but it is increasingly becoming a pediatric health problem and this has been linked partly with the global increase in obesity (OB) and physical inactivity^3^. It has been established that T2DM is a highly inheritable disease with about 90% of the youth affected having a family history of either first-or-second-degree relative being previous sufferer^4^. Other recognized risk factors include ethnicity, hypertension, dyslipidemia and acanthosis nigricans^5^.

One of the recognized key risk factors of T2DM in the pediatric population is childhood overweight (OW) or OB^6^. Although OB is known to be associated with several cardiometabolic disease (CMD)^7^,^8^, recent studies have clearly shown localization or distribution of adipose tissue to be more closely related to these diseases than OB per se^9^,^10^. These findings have renewed interest of the scientific community in the study of adipose tissue distribution and its relative effect on health^11^,^12^. Previous studies in adolescents have shown that central adiposity was more closely associated with the risk of T2DM than general adiposity^13^,^14^,^15^. In a study of Chinese adolescents aged 13–15 years^10^, participants with abdominal OB as measured by waist-to-height ratio (WHtR) were found to have significantly higher fasting blood glucose (FBG) compared to their non-obese peers. A systematic review and meta-analytic study^16^ also reported that waist circumference (WC) and WHtR had excellent discriminating power for central fat and CVD risk factors in children and adolescents.

Nigeria, like many developing countries is undergoing rapid socioeconomic transition, and this creates the need to empirically determine how these changes affect the adolescent population in areas of chronic disease risks and body weight disorders generally. However, the measure of fat that is hazardous with respect to its effect on diabetes mellitus remains to be clearly elucidated. Thus, the purpose of this study was to examine the predictive capacity of these anthropometric body fat indicators to detect risk of T2DM among Nigerian adolescents. The study also determined the association of central obesity, that is, WC and WHtR and general adiposity, that is, body mass index (BMI) with risk of T2DM in Nigerian adolescents. In a resource limited setting, the use of anthropometric data to predict the risk of T2DM will be cost-effective modality. The ability of each body fat proxy to discriminate between euglycemia and risk of T2DM among participants would be of public health significance.

## Methods

### Study Design and Sample

This is a cross-sectional study comprising apparently healthy secondary school girls and boys aged 11–19 years drawn from four secondary schools in Kogi East Senatorial District, Kogi State of Nigeria. The Lorentz formula for population greater than 10000 was used to calculate the minimum size, with a prevalence of 1.9% obtained from the pilot test for the present study as recommended^17^. The minimum size calculated was 380 participants which was increased to 418 to account for possible attrition and enhance representativeness. The study was conducted during the months of September to December, 2019. Participants were selected using systematic sampling procedure. In this procedure, every 4^th^ student beginning from a particular number on the class register was selected to participate in the study. Inclusion criteria were: Secondary school adolescents between ages 11–19 years, not diagnosed for diabetes mellitus, and whose parents gave consent. Exclusion criteria included sick adolescents and those who had breakfast the day of the test.

### Data Collection

Physical characteristics of participants were measured using standard procedures^18^. Body mass and stature were measured indoors with the aid of an electronic weighing scale (Seca digital floor scale, Sec-880; Seca, Birmingham, UK) and a portable stadiometer (Model Sec-206; Seca, UK). Participants' body mass index (BMI) was computed and expressed as weight in kilograms divided by stature in meters (kg.m^-2^). Both the triceps and medial calf skin-fold thickness was measured on the right side of participants' bodies with the aid of the Harpenden Skinfold Calipers (Creative Health Products, MI, USA). All measurements were taken thrice and the median of the three readings recorded. The revised regression equations for black children, were used to estimate percent fat^19^. Waist circumference (WC), an estimate of abdominal fat, was measured with a retractable metal tape (Creative Health Products, MI, USA) at the level of umbilicus and midway between the lower rib margin and the iliac crest. Readings were taken at the end of a quiet expiration to the nearest 0.1cm. Two measurements were taken and the average score recorded. The waist-to-height ratio (WHtR) was calculated by dividing the WC in centimetres by stature in centimetres.

A twelve-hour overnight fasting blood glucose (FBG) was obtained from capillary blood samples analyzed with a CardioCheck Plus Analyzer (CCPA) (PTS Diagnostics, Indianapolis, IN, USA). Participants were asked to sit down for at least ten minutes after which they took their turns for the test. The middle finger was cleaned with an alcohol wipe after which a gentle pressure was applied to the finger with lancet and the finger pricked at the center. Thereafter, gentle pressure was applied to the finger to produce a large drop of blood which was dispensed on the test strip. Test results were then displayed on the analyser within 90 seconds. The CCPA is a valid and reliable instrument for analysing blood glucose (GLU)^20^. Details of the protocol have been previously described^21^.

### Definition of metabolic risks

The criteria used for defining the metabolic risk abnormalities and body fat indicators used in this study were those recommended by internationally recognized authorities and health agencies. The standards used for FBG (≥5.6mmo/L) and WC (90^th^ percentile for age and sex) are those recommended by the IDF^22^. The cut-points used for WHtR (0.46) and BMI (95^th^ percentile for age and sex) were those recommended by Meng et al.^23^ and The Cooper Institute 18 respectively.

### Pilot Test

Before the commencement of the study, a pilot test was conducted to refine test administration procedures and determine precision of the instruments for data collection. Forty adolescent girls and boys ranging in age from 11–18 years that did not form part of the sample participated in the test. All measurements were taken according to standard procedures, and the Cronbach Alpha Coefficient was calculated to determine the reliability. In all cases, the alpha coefficients ranged from 0.752–0.87, indicating good internal consistency^24^

### Data Analysis

All analyses were conducted using the Statistical Package for the Social Sciences (Version 20, SPSS Inc, Chicago, IL, USA) at a probability level of 0.05. Data were checked for normality before analyses with the Kolmogorov Smirnov test. Complete data for all variables were available for 403 out of 418 adolescents, a compliance rate of 96%. Descriptive data were expressed as means ± SDs, frequencies and percentage distributions. Significant differences between adiposity categories for physical characteristics and FBG were determined using independent samples t-test. Predictive performances of body fat proxies for risk of T2DM were determined through the receiver operating characteristics curve analysis (ROC) with 95% confidence interval (95%CI). Accurate threshold values for detecting T2DM risk were determined through area under curve (AUC) values, sensitivity and specificity. The values of AUC were interpreted using a set of guidelines^25^: An AUC of 0.5 = no discriminatory power; >0.9 = excellent; 0.8–0.9 = good; 0.7–0.8 = moderate; < 0.7 = poor. Zero-order correlation coefficients were used to assess the relationships among the variables. Multivariate regression models assessed the independent association of body fat indices with T2DM risk.

### Ethical Clearance

All tests were conducted from 9: 00am -12 noon in accordance with the principles of Helsinki Declaration after prior approval was received from the Ethical Review Committee of The College of Health Sciences (ID: COHS/02/25/2020), Kogi State University, Nigeria on the 5^th^ of May, 2019. Written informed consent of parents and assent of minors were obtained before data collection.

## Results

**Figure 1:**
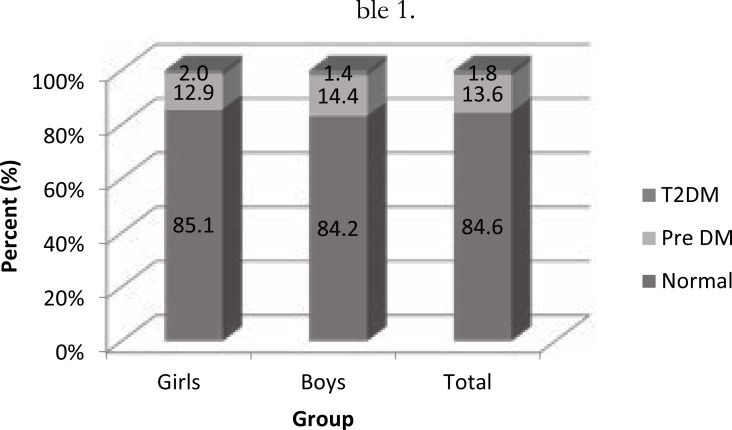
Distribution of participants by glycemic profile

**Figure 2:**
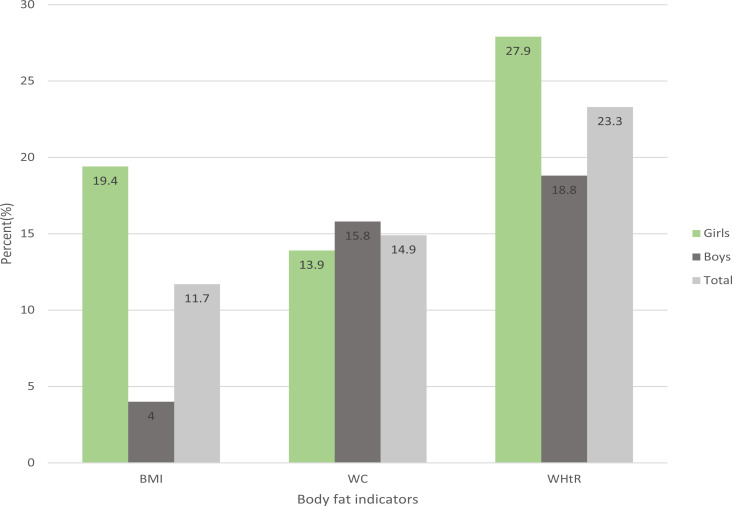
Prevalence of obesity by different body fat indicators

The general characteristics of the sample are presented in Table 1 stratified according to gender. The glycemic profile of participants (combined) indicated that 84.6%, 13.6% and 1.8% were normal, pre-diabetic and diabetic respectively. Details of the gender-specific prevalence are shown in Figure 1. Prevalence of OB determined by each body fat indicator is presented in Figure 2. As shown, both WHtR and WC displayed higher proportions of obesity (23.3% and14.9%) than BMI (11.7%) in both genders combined. On the average, girls were significantly heavier (*p*<0.016), fatter (*p*<0.001), displayed higher BMI (*p*<0.001), WC (*p*=0.001) and WHtR (*p*<0.001). There were no significant gender differences in age (p=0.558), stature (*p*=0.193) and FBG (*p*=0.157) as indicated in Table 1.

**Table 1:** General characteristics of participants stratified by Gender (n = 403)

Variable	Combined (n=403)	Girls (n=201)	Boys (n=202)	t-value	p-value
Age (y)	14.7 ±2.3	14.8 ± 2.3	14.7 ± 2.2	0.586	0.558
Stature (cm)	160.2 ±9.8	159.6± 7.1	160.9 ± 11.9	0.303	0.193
Body mass (kg)	53.1 ± 12.5	55.5 ±12.1	50.8 ± 12.5	3.784	0.016
Body fat (%)	15.5 ± 7.0	19.8 ± 7.4	11.2 ± 2.9	15.31	<0.001
BMI Kg.m^-2^	20.5 ± 3.5	21.7 ± 4.0	19.3 ± 2.6	6.929	<0.001
WC (cm)	65.8 ± 8.8	67.2 ± 9.4	64.4 ± 8.0	2.273	0.001
WHtR	0.41 ± 0.1	0.42 ± 0.1	0.40 ± 0.1	1.93	<0.001
FBG (mmol/L)	5.1 ± 0.7	5.0 ± 0.7	5.1 ± 0.7	0.401	0.157

The ROC curve analyses are presented in Table 2. In boys the areas under curve were significantly greater than 0.5 for both WC (*p*<0.001) and WHtR (*p*<0.001) ranging from 0.767 t 0.807. But the AUC for BMI (*p*=0.342) was not significantly different from chance as diagnostic tests for T2DM risk. The optimal threshold for WHtR was 0.40 and that for WC was 61.8 cm. For both variables, the sensitivities were high while the specificities were low. That is, these tests were good at identifying most of the adolescents at risk of T2DM but also missing out a few participants with the risk. In the case of girls, the areas under curve were not significant (p>0.05) for all the body fat indices. Details of the results are presented in Table 2.

**Table 2:** Receiver Operating Characteristic curve analysis for risk of Type 2 Diabetes Mellitus (n=403)

Gender	Variable	AUC	95%CI	Cut- point	Sensitivity	Specificity	p-value
Girls	WC	0.548	0.454–0.642	65.5	0.556	0.436	0.366
	WHtR	0.545	0.448–0.642	0.42	0.556	0.388	0.397
	BMI	0.522	0.424–0.620	21.8	0.500	0.430	0.676
Boys	WC	0.767	0.693–0.841	61.8	0.816	0.333	<0.001
	WHtR	0.807	0.736–0.877	0.40	0.816	0.327	<0.001
	BMI	0.455	0.355–0.555	18.5	0.469	0.601	0.342

The zero-order correlation coefficients between body fat indicators and the dependent variables showed that the relationships were significant only in boys, and can be described as weak to moderate. The strongest relationship was that between FBG and WHtR (r=0.502, p<0.001), followed by WC (r=0.419, *p*<0.001). Among the body fat indices, the strongest relationship was between WHtR and WC in both boys (r=0.834, *p*<0.001) and girls (r=0.953, *p*<0.001) (Table not shown).

Results of the multivariate regression models assessing the extent to which the body fat proxies could predict FBG after controlling for age are presented in Table 3. In boys, age (p=0.009) explained 3% of the variance in FBG in step 1. The addition of body fat proxies in step 2 increased the total variance cumulatively to 29%, thus indicating that body fat indices explained additional variance of 26% after controlling for the covariate. Waist-to-height ratio(*β* =0.599, p<0.001) and BMI (*β*=-0.175, p=0.045) were the only significant predictors in step 2. In the model for girls, only age was the significant predictor, explaining 5% of the variance in FBG in step 1 (*p*=0.001). Addition of body fat indices in step 2 improved the variance by only 1%.

**Table 3:** Adiposity indices as predictors of FBG adjusted by age (n=403)

		Model1			Model2		
Group	Variable	r^2^	*β*	*P*	r^2^	*β*	*p*
Girls	Age	0.052	0.230	0.001	0.064	0.249	0.006
	WC	-	-	-	-	0.133	0.603
	WHtR	-	-	-	-	-0.186	0.430
	BMI	-	-	-	-	0.069	0.465
Boys	Age	0.034	0.184	0.009	0.285	0.243	0.012
	WC	-	-	-	-	-0.147	0.411
	WHtR	-	-	-	-	0.599	<0.001
	BMI	-	-	-	-	-0.175	0.045

## Discussion

Findings from the present study indicate that: prevalence of the risk of T2DM is relatively high compared to previous studies in adolescents, and it is slightly higher in girls; the association between FBG and body fat indicators was weak to moderate; and WHtR demonstrated the highest discriminating power in detecting risk of T2DM in boys but not girls.

The combined prevalence of pre-diabetes of 13.6% and T2DM of 1.8% documented in this study is higher than the values of 3.1% and 0.2% pre-diabetes and T2DM respectively reported for Chinese adolescent boys^26^. The prevalence rates of 16.1% for pre-diabetes and 0.6% for T2DM reported for Ivorian adolescents are comparable with our results^27^. Evidently, the prevalence of blood glucose abnormalities tended to be high among African adolescents.

Findings from this study show that the relationship between the dependent variable and the body fat indices were weak to moderate, with that between FBG and WHtR being the strongest. Recently, WHtR was shown to be more closely linked to childhood and adolescent morbidity than BMI^28^. The strongest correlation among the body fat indices was that between WHtR and WC, implying both independent variables (representing central adiposity) are better surrogates of body fat than BMI and body fat. The 29% variance in the risk of T2DM explained by body fat indicators in boys shows that adiposity, especially central adiposity is an important predictor of diabetes^7^,^13^. The unexplained variance of 71% may be due to other variables such as hypertension, blood lipids, and others^5^ which were not considered in this study. The fact that in this study the prevalence of OB determined by WHtR and WC were higher than any other body fat indicator in both genders (Figure 2) is an indication that these central fat indices are more important explanatory variables than total fat for risk of T2DM in this study.

In the present study, WC, WHtR and BMI were compared to determine which body fat proxy best predicted risk of T2DM. The anthropometric parameter of central OB, WHtR was found to predict T2DM better than generalized OB (BMI) in boys. WHtR demonstrated the best utility for screening T2DM among boys. But none of the body fat indicators was a significant predictor of T2DM in girls. The findings in boys are in agreement with previous studies in youth.^14^,^21^ The optimal thresholds for WHtR of 0.40 and that for WC of 61.8 cm are comparable to those reported in previous studies in adolescents^22^,^29^.

Girls' results were unexpected. The explanation that can be provided for now is that these fat indices are not important explanatory variables for T2DM risk among this cohort of girls. These independent variables explained only 1% of the variance in T2DM, implying that other variables including, hypertension, blood lipids, physical inactivity, heredity and depressive symptoms could be more important in predicting diabetes in this sample of girls^30^. Another possibility may be the preponderance of post-pubertal girls in the study sample (age range = 11–19 y). This category of girls is highly predisposed to gluteofemoral fat deposition which is known to have a protective role in the development of cardiometabolic disease^31^. This might have lowered the sensitivity of other fat indicators to detect T2DM in girls. The thresholds of body fat proxies for identifying risk of FBG abnormalities in boys documented in this study are generally lower than the reference standards. This may lead to underestimation and misclassification of both central and general OB in this population. This clearly indicates that these international standards are not suitable for African youth. A plausible reason may be the exclusive use of samples from industrialized countries in developing these standards. Smaller body frame of African youth may be another reason. Added to these, ethnicity is known to influence anthropometric indicators of body fat and individual components of MS in adolescents^32^.

A limitation of this study was the cross-sectional design used as it precludes confirmation of cause-and-effect relationship between the variables. Nevertheless, a major strength of this study is the use of ROC which provided population-specific thresholds for the anthropometric body fat indicators for detecting risk of T2DM.

## Conclusion

Our findings suggest that central fat is more effective tool than total fat for identifying the risk of T2DM among Nigerian adolescent boys. But none of the fat indicators was effective in identifying T2DM risk in girls. Indeed, WHtR should be the first tool of choice in screening for T2DM risk among Nigerian adolescent boys, while WC is a reasonable alternative. These body fat proxies demonstrated good capacity to detect risk of T2DM.

## Recommendations

Population-based studies on diagnostic accuracy of anthropometric body fat indicators in identifying risk of diabetes mellitus in African subjects are warranted. This will make it possible to establish cut-point values for African adolescents. Identifying and developing strategies for screening high risk adolescents should be considered an important public health goal. The public health challenge therefore lies in the use of appropriate body fat indicators by stakeholders in pediatric health promotion to identify adolescents at risk of T2DM for early prevention program.
